# The Brighton Hospitals

**Published:** 1904-10-08

**Authors:** 


### THE BRIGHTON HOSPITALS.

### APPOINTMENT OF A CENTRAL HOSPITAL BOARD.

A large and influential meeting was held at Brighton on the 3rd instant under the presidency of the Mayor, Alderman E. M. Marx, who for some months has been working assiduously to unite all the hospital authorities in Brighton and the district, with the objects of preventing overlapping, diminishing expenditure, controlling the abuse of the hospitals, and securing the maximum benefit for the inhabitants from the bed accommodation provided by all the hospitals concerned. The Mayor in an excellent speech explained the various meetings, conferences, and negotiations which had taken place during the past few months, and stated that the following was the

#### Result of the Inquiries.

The net result of the work which had been done up to the present might be summarised as follows:—

(1) Saving to the charitable public of a new institution at a cost of £24,000.

(2) Abolition of overlapping in gynæcological work.

(3) Establishment on a sound basis of the valuable "maternity school."

(4) Overlapping of treatment of diseases of children much minimised, because the cases formerly dealt with in the Grant Block would have to go to the Children's Hospital, the work of which would in the future be far more useful than in the past, as there would now be no room for slight cases somewhat unsuited to indoor hospital treatment.

(5) The creation of a long-desired and necessary special department for gynæcology.

(6) The adjustment of the honorary medical work in the Sussex County Hospital and Women's Hospital on a satisfactory basis, and the establishment of even more cordial relations than in the past between the medical men of the two institutions.

They ought not to, and could not, rest content with even these great gains. In order to consolidate and make permanent the results obtained, and even to extend them so far that none of the defects should continue to exist, further action was required, and he ventured to believe that in the proposals which he had to make that day they would find a solution of the problems before them.

The Mayor eventually moved "that this meeting of governors and supporters of the local medical charities is of opinion that a Board, to be called the Brighton, Hove, and Sussex Hospital Board, should be appointed with the following objects:—

(1) To obviate overlapping of local medical charities.

(2) To promote economy of administration, particularly in connection with capital outlay on buildings; and

(3) To prevent the abuse of medical charities by persons not proper objects for charitable assistance."

This resolution dealt with the defects which he touched upon in his opening, and set forth clearly the end in view. A Central Board constituted by a gathering such as that could not fail to enjoy the confidence of the charitable public, and the considered results of its deliberations must rightly have great weight. But it must not be forgotten that the only power which this body could enjoy was the inherent power of carefully weighed conclusions based on adequate investigation and expressed in a judicial and conciliatory spirit in due season by persons who enjoyed the public confidence. There was not and there could not be a thought of compulsion or dispossession. Success would depend, as he believed the results of the past had depended, upon the fact that the principles put forward were sound and for the public benefit, and would be advanced in a temperate and conciliatory manner with due regard for the rights and privileges of all interested. (Loud applause.)

The Mayor of Hove, heartily agreeing with the scheme, seconded the resolution without comment.

After some animated discussion in which differences of opinion were manifested, the Mayor's resolution was carried by 60 votes to 20. The Rev. A. R. C. Cocks moved, Mr. Leonard Holmes seconded, and it was resolved that, "for the attainment of these objects, the Board be authorised to take such steps as they may think advisable, including—

(1) The promotion of cordial relations between the Board and the Managing Committees of the several hospitals, and between such Committees, with a view to the adoption by the latter of any suggestions which the Board may consider likely to promote their objects.

(2) The compilation of statistical information as to the apportionment of different classes of patients between the several hospitals; and

(3) Similar information as to the cost of maintenance and administration, particularly in respect of capital outlay on buildings.

(4) The institution and carrying out of negotiations with, and between, the governing bodies of any Charities which might be more economically administered by mutual arrangement.

(5) The raising of subscriptions for meeting any expenses of the Board.

(6) Consideration of the question of the continuance of the Board as a properly constituted public body, and for the election or appointment of its members, and the definition of its aims and powers, including power to accept the responsibility for and management of any institution the governing body of which may be desirous of placing it under the control of the Board.

It was further resolved on the motion of Mr. H. J. Verral that 71 gentlemen, including the Mayors and the Vicars of Brighton and Hove *ex officio* should be invited to become the first members of the Board, with power to co-opt not more than five additional members, and to hold office until December 31st, 1905, or such other date as may be appointed by a resolution of the majority of those attending a meeting of those summoned that day, such meeting to be convened at the request of the Board by the Chairman or the Vice-Chairman of such Board. An amendment by Mr. H. Wakefield recommending that the Mayor should communicate with each institution asking them to send delegates to form the Board, the number of delegates from each institution to be the subject of further consideration, having been negatived, Mr. Verrall's resolution was agreed to. A subscription list was then opened, and the meeting closed with the following vote of thanks to the Mayor, moved by Mr. A. C. Lee and seconded by Dr. Willoughby Furner:—

That this meeting records its appreciation of the signal service rendered by the Mayor of Brighton (Alderman E. M. Marx) in bringing to a successful issue the preliminary negotiations which have been carried on by him during the past nine months with the authorities of certain of the local hospitals, the result of which has been to prepare the way for the adoption of a scheme for the co-ordination of the medical charities of the district.

